# EDUCATION AS A MEDIATOR OF GENDER DIFFERENCES IN COGNITION IN OLDER ADULTS

**DOI:** 10.1093/geroni/igad104.2469

**Published:** 2023-12-21

**Authors:** Judith Rijnhart, Matthew Valente, Almar Kok, Martijn Huisman

**Affiliations:** University of South Florida, Tampa, Florida, United States; University of South Florida, Tampa, Florida, United States; Amsterdam University Medical Center, Amsterdam, Noord-Holland, Netherlands; Amsterdam University Medical Center, Amsterdam, Noord-Holland, Netherlands

## Abstract

Although dementia is more prevalent in women than in men, studies on the socio-cultural mechanisms that cause and sustain gender differences in dementia remain scarce. Historic gender differences in education may explain why older women are at higher risk of dementia nowadays. This study aimed to investigate education as a mediator of gender differences in cognitive function, as measured with the Mini-Mental State Examination (MMSE). We used data from the Longitudinal Aging Study Amsterdam. The analytical sample consisted of 2,909 older adults aged 55-85, of whom 1,601 were women and 1,506 were men. We used causal mediation analysis to estimate total, indirect, and direct effects of gender on MMSE. Effect estimates were adjusted for confounding by age, parental education, and childhood adversity. The total effect was -0.60 (95% CI: -0.90; -0.32), indicating that women on average had a 0.60 lower MMSE score than men. The indirect effect was -0.84 (95% CI: -1.11; -0.59), indicating that women on average had a 0.84 lower MMSE score compared to men, because women completed fewer years of education. The direct effect was 0.24 (95% CI: 0.04; 0.44), indicating that if women had the same number of education years as men, their MMSE score would on average be 0.24 points higher than the average MMSE score in men. These findings indicate that education is an important mediator of gender differences in cognitive function. Future studies are needed to explore whether gender differences in cognitive function decrease as gender equality in education increases.

